# An ethical issue: nurses’ conscientious objection regarding induced abortion in South Korea

**DOI:** 10.1186/s12910-020-00552-9

**Published:** 2020-10-27

**Authors:** Chung Mee Ko, Chin Kang Koh, Ye Sol Lee

**Affiliations:** 1 College of Nursing, Sungshin Women’s University, 55, Dobong-ro 76ga-gil, Gangbuk-gu, Seoul, 01133 Republic of Korea; 2 College of Nursing, The Research Institute of Nursing Science, Seoul National University, 103 Daehakro, Jongrogu, Seoul, 03080 Republic of Korea; 3 College of Nursing, Seoul National University, 103 Daehakro, Jongrogu, Seoul, 03080 Republic of Korea

**Keywords:** Conscientious objection, Abortion, Nursing ethics, Women’s health, Legal reform

## Abstract

**Background:**

The Constitutional Court of South Korea declared that an abortion ban was unconstitutional on April 11, 2019. The National Health Care System will provide abortion care across the country as a formal medical service. Conscientious objection is an issue raised during the construction of legal reforms.

**Methods:**

One hundred sixty-seven perioperative nurses responded to the survey questionnaire. Nurses’ perception about conscientious objection, support of legislation regarding conscientious objection, and intention to object were measured. Logistic regression was used to explore the factors associated with support of the legislation and the intention to conscientiously object.

**Results:**

Only 28.8% of the responding nurses were aware of health care professionals’ conscientious objection. The majority (68.7%) felt that patients’ rights should be prioritized over health care professionals’ conscientious objection. On the other hand, 45.8% supported the legislation on conscientious objection to abortion, and 42.5% indicated a willingness to refuse to participate in an abortion case if conscientious objection was permitted. Religion, awareness of conscientious objection, and prioritizing of nurses’ right to conscientious objection were significantly associated with supporting the legislation. Moreover, religion and prioritizing nurses' rights were significantly associated with the intention to conscientiously object.

**Conclusions:**

This study provides information necessary for further discussion of nurses’ conscientious objection. Nursing leaders, researchers, and educators should appeal to nurses and involve them in making policies that balance a women's right to non-discrimination and to receiving appropriate care with nurses' rights to maintain their moral integrity without compromising their professional obligation.

## Background

On April 11, 2019, the Constitutional Court of South Korea ruled that the criminalization of abortion is unconstitutional [1]. The Constitutional Court ruled that women’s self-determination would take priority during the first 22 weeks of pregnancy [2]. According to Articles 269 and 270 of Chapter XXVII, “Crimes of Abortion” in the Criminal Act, a woman who procures her own miscarriage or a healthcare provider such as an obstetrician or midwife who performs an abortion for a woman shall be punished by a fine or imprisonment [3]. The Constitutional Court ruled these two articles as constitutional discordance and gave the National Assembly until the end of December 2020 to revise the law [2]. Therefore, the National Assembly is working on reforming related provisions of the Criminal Law and the Mother and Child Health Act. The Government is also working to support law making and to align policies and regulations implementing the new ruling while researching public opinions. Throughout this process, the National Health Care System will provide abortion care across the country as a formal medical service. This indicates that many health care providers in hospitals and clinics who have never been involved with abortion because it was performed covertly in a limited number of clinics will face abortion cases for the first time in their workplaces. Nationally, the number of annual abortions covertly performed is estimated at 50,000 cases, 90% of which are surgical abortions [4].

Conscientious objection (CO) is an issue raised during the construction of legal reforms. Currently, Paragraph 1 of Article 15 (Prohibition against Refusal to Provide Medical Services) under the Medical Service Act states: “Medical personnel or the founder of a medical institution shall not upon receiving a request for medical treatment or assistance in childbirth refuse such a request without good cause” [5]. Under this legislation, health care providers’ refusal to perform an abortion is banned [6]. The Ministry of Health and Welfare virtually stated its opposition to healthcare providers’ CO considering health care access at an official meeting held at the National Assembly [7]. However, physician groups such as the Korean Society of Obstetrics and Gynecology and Korean Association of Obstetrics and Gynecology have announced their position in support of physicians’ right to CO and have asked for new abortion laws that include provisions ensuring the right of healthcare workers to refuse abortions [8]. On the other hand, nurses and other health professional groups have not provided their official position about CO.

In some countries, the law contains provisions for the right of CO to abortion. For example, "Discussion" section of the Abortion Act in the UK states that health care providers may refuse to participate in any treatment based on his or her CO [9]. The Act recognizes the right to CO status related to abortion of which provision is ethically controversial and on which many people have strong views [10]. On the other hand, other countries do not legally grant the CO of health care providers [11]. For example, Sweden does not have legislation for CO under its Abortion Act, and the Swedish Parliament has rejected including a clause for CO [12]. Moreover, the World Health Organization has expressed reservations about CO because it may delay the delivery of health care services and place a woman’s health at risk [13].

There have been arguments for and against nurses’ CO. An argument for CO is that it is a human being’s fundamental moral right. Article 9 of the European Convention on Human Rights states that everyone has the right to freedom of thought, conscience, and religion [14]. Permitting CO avoids moral distress, which might occur when a nurse performs certain acts inconsistent with his or her beliefs [15]. Nurses who declared a CO in their practice stated that this was “based on one’s conscience-based perception of morality” [16, p.1343]. Moreover, CO protects health care providers by crediting the individual’s conviction, and it supports health care providers in following their religious beliefs or religious law against providing abortion-related care [17]. An argument against CO in nursing is that it might negatively influence access-of-care or appropriate health care services [18]. Patients may have fewer options, and there is concern about the possible widespread practice of CO [17]. In addition, co-workers may see objectors as leaving unpleasant tasks to their colleagues [15].

Fleming et al. conducted a systematic review and identified 23 broad reasons and 116 narrow reasons in 4 categories, namely moral, practical, religious, and legal reasons [17]. Of the narrow reasons, 70% were for and 30% against CO in abortion care by midwives and nurses. Considering these reasons, balancing nurses’ right to CO and the patient’s right to receive appropriate care is a challenge in nursing [18]. We need to carefully evaluate what treatment boundaries we allow conscience to determine when considering social and health care environments [10]. However, in South Korea, no studies have focused on nurses’ CO to abortion care, although they now face an era of legal abortion as a formal medical service. Moreover, CO policy will have great impact on perioperative nurses [19].

Perioperative nurses have the duty of providing care relating to the surgical terminations of pregnancy, which is one ethical dilemma they may face in an operation [19]. Perioperative nurses prepare the theatre for procedures and assist in termination [19]. They also provide psychosocial care for women needing an abortion and for their significant others [20, 21]. In terms of CO, according to the American Nurses Association guidelines, scrub and circulating nurses may object to providing instruments for the termination of a pregnancy when it causes moral distress [22]. The Association for Perioperative Practice in the UK also supports nurses’ right for CO regarding surgical abortion based on the Human Fertilization and Embryology Act of 1990 [19].

The purpose of this study was to explore perioperative nurses’ attitudes towards CO regarding abortion. The specific aims were (1) to examine whether they were aware of CO and how they prioritize between nurses’ right to CO and patients’ right to get an abortion, (2) to describe agreement with legislation of nurses’ CO, and (3) to explore nurses’ intention to assert CO.

## Methods

### Study design

This was a cross-sectional descriptive study utilizing a self-report survey method. The data was collected in October and November 2019**.**

### Measurements

Prior to inquiring about the nurses’ perceptions of and ideas about CO, we provided information about CO regarding abortion care because this was unfamiliar to the majority of the nurses. We provided a brief description based on the previous literature [9–11]. Each concept was measured using one question we developed based on the literature. Prior to the survey, we verified face validity by having ten nurses review the questions to assess their clarity, comprehensibility, and appropriateness.

1. Support of legislation on conscientious objection to abortion

We asked nurses about whether they support the legislation ensuring nurses’ rights to CO: “Do you support that nurses’ right to CO should be ensured under the law?” The answer options were “support,” “oppose,” and “neither support nor oppose.”

2. Intentions to assert conscientious objection

The question regarding intention to assert CO was: “Would you object to participating in abortion care if CO for nurses were to be allowed by law?” Moreover, three situations were given [11], namely “abortion because of foetal problems within 22 weeks,” “abortion because of rape within 22 weeks,” and “abortion because of unwanted pregnancy within 22 weeks.” We posited a 22-week duration in which to allow abortions, which is in accordance with the judgement of the Constitutional Court on April 11, 2019. The answer choices were “I will object,” “I will not object,” and “I do not know.”

3. Awareness of CO and opinions about the conflict over nurses’ CO

The question used to assess nurses’ awareness of CO was “Prior to this survey, were you aware of health care workers’ CO?” The answers were measured on a five-point Likert Scale with the following options: 1 = “fully unaware,” 2 = “unaware,” 3 = “neither aware nor unaware,” 4 = “aware,” and 5 = “fully aware.” Furthermore, to ascertain their opinion about the conflict between the rights of nurses and those of patients, we asked: "If there is a conflict between nurses’ right to CO and patients’ right to have an abortion, which do you think should take priority?" [10]. The answers allowed were “nurses’ right to CO,” “patients’ right to have an abortion,” and “I do not know.”

### Data analysis

We performed logistic regression to explore related factors and their odds ratios regarding nurses’ support of legislation to ensure CO and their intention to assert CO. For the logistic regression model of the nurses’ support of the legislation, we coded the nurses’ support as a dichotomous variable. We coded “oppose” and “neither support nor oppose” as 0, and “support” as 1.

In addition, for the logistic regression model, we coded nurses’ intention to assert CO as a dichotomous variable. We assigned “0′ in all cases where they did not choose “will reject” to all three items. These three items were “abortion because of foetal anomaly within 22 weeks,” “abortion because of rape within 22 weeks,” and “abortion because of unwanted pregnancy within 22 weeks.” We assigned “1” to all cases in which the respondent choose “will reject” to at least one of the three items. We utilized crude odds ratios and confidence intervals to show the association. The significance level was 0.05.

## Results

### Study participants

This study included 167 perioperative nurses who were working in an operating department, including the preoperative area, operating theatres, and post-anaesthesia care unit, in a large tertiary care university hospital in the Seoul metropolitan area of South Korea. The total number of perioperative nurses was 203. Thus, the response rate was 82%. The mean age was 34.4 years, ranging between 22 and 57 years (Table 1). Among the 167 perioperative nurses, 152 were female, and 15 were male. Less than half (47.3%) reported being religious. The types of religion were Protestant, Catholic, and Buddhist. No other religions were reported. The average clinical experience was 11.03 years. In terms of clinical roles, the majority (62.9%) were operating room nurses. Other roles were recovery room nurses (15.6%), anaesthesia nurses (14.4%), and physician assistants or surgical assistants (7.2%).

**Table 1:** Characteristics of study participants (N = 167)

	Frequency (%)	Mean (SD)
Age		
30 years or younger	64 (38.3%)	34.40 (8.63)
31–40 years	66 (39.5%)	
41 years or older	37 (22.2%)	
Gender		
Female	152 (91.0%)	
Male	15 (9.0%)	
Marital status		
Single	97 (58.1%)	
Married	70 (41.9%)	
Religion		
No religion	88 (52.7%)	
Protestant	47 (28.1%)	
Catholic	25 (15.0%)	
Buddhist	7 (4.2%)	
Religion-importance		
No religion or not important at all	89 (53.3%)	1.91 (1.09)
Not very important	21 (12.6%)	
Somewhat important	39 (23.4%)	
Very important	18 (10.8%)	
Clinical experience (years)		
10 years or less	91 (54.8%)	11.03 (8.84)
More than 10 years	75 (45.2%)	
Clinical role		
Operating room nurse	105 (62.9%)	
Recovery room nurse	26 (15.6%)	
Aesthesia monitoring nurse	24 (14.4%)	
Physician assistant/surgical assistant	12 (7.2%)	
Awareness about CO		
Fully aware/aware	48 (28.7%)	2.68 (1.10)
Fully unaware/unaware/neither aware nor unaware	119 (71.3%)	
Resolving conflicts between nurses’ right to CO and patients’ right to receive abortion care		
Nurses’ rights prioritized	35 (21.1%)	
Patients’ rights prioritized	114 (68.7%)	
I do not know	17 (10.2%)	
Legislation for nurses’ rights for CO to abortion		
Support	76 (45.8%)	
Oppose	38 (22.9%)	
Neither support nor oppose	52 (31.3%)	

*CO* conscientious objection

In terms of CO, the mean score of awareness of CO was 2.68 (SD = 1.10). Just 48 nurses (28.8%) reported being “fully aware” or “aware” of the CO of nurses and physicians. The majority (68.7%) felt that patients’ rights should be prioritized over CO.

### Legislation on conscientious objection to abortion

Among the perioperative nurses, 45.8% supported the legislation on CO to abortion, 22.9% opposed it, and 31.3% neither supported nor opposed it. In the logistic regression, age, gender, marital status, clinical experience, and clinical role were not significantly associated with the nurses’ support of the legislation on CO to abortion (Table 2). On the other hand, the Protestant religion was significantly associated with support. Nurses who were Protestants were more likely to support the legislation than those who had no religion (OR = 2.471; 95% CI 1.189–5.133, *p* = 0.015). Moreover, the perceived importance of religion was also associated with their support of the legislation on CO to abortion (OR = 0.400, 95% CI 0.207–0.773, *p* = 0.006).

**Table 2:** Support of legislation confirming health care professionals’ right to assert conscientious objection to abortion (N = 167)

	Odds ratio	95% confidence interval	*p* value
Age (years)	1.015	.979–1.052	.411
Gender			
Female	.717	.247–2.077	.540
Male	Reference		
Marital status			
Married	.853	.458–1.587	.615
Single	Reference		
Clinical experience (year)	1.010	.975–1.046	.580
Clinical role			
Recovery	1.502	.626–3.603	.362
Anaesthesia	1.173	.481–2.861	.726
PA/SA	2.773	.785–9.788	.113
Op room	Reference		
Religion			
Protestant	2.471	1.189–5.133	.015
Catholic	1.059	.427–2.625	.902
Buddhist	2.118	.446–10.050	.345
No religion	Reference		
Religion (importance)	.400	.207–.773	.006
Awareness about CO			
Fully aware/aware	2.042	1.033–4.035	.040
Fully unaware/unaware/neither aware nor unaware	Reference		
Resolving conflicts between nurses’ right to CO and patients’ right to receive abortion care			
Nurses’ rights	6.750	1.762–25.853	.005
Patient’s rights	.563	.201–1.574	.273
Do not know	Reference		

CO: conscientious objection

On the other hand, the odds ratio of the awareness of CO was 2.042, with a 95% confidence interval of 1.033–4.035 (*p* = 0.040) (Table 2). In addition, the odds ratio of the nurses who answered “nurses’ rights take priority” in the case of a conflict between nurses’ rights to CO and patients’ right to receive nursing care for abortion was 6.750 with a 95% confidence interval of 1.762–25.893 (*p* = 0.005) where the reference group was the nurses who answered “I do not know.”

### Nurses’ intentions to assert conscientious objection.

There were three items about the intention to assert CO based on the different reasons for having an abortion (Table 3). In case of abortion because of foetal problems, the percentage answering that they would object was 21.6%; it was 22.8% in cases of abortion because of rape; and it was 31.3% in cases of abortion because of unwanted pregnancy.

**Table 3:** Intentions to assert conscientious objection (N = 167)

	Conscientious objection		
	Will object	Will not object	Do not know
Abortion because of foetal anomaly within 22 weeks	36 (21.6%)	105 (84.4%)	26 (15.6%)
Abortion because of rape within 22 weeks	38 (22.8%)	114 (68.3%)	14 (8.4%)
Abortion because of unwanted pregnancy within 22 weeks	52 (31.3%)	67 (40.4%)	47 (28.3%)

The number of those reporting a willingness to refuse being involved in the care of those receiving abortions for at least one of the three following reasons—foetal anomaly, rape, or unwanted pregnancy—was 71 (42.5%). From the logistic regression, protestant perioperative nurses were more likely to report that they would assert CO relative to those having no religious preference as the reference group (Table 4). The odds ratio was 2.209 with a 95% confidence interval of 1.073–4.549. The perceived importance of religion was also a significant factor related to the intention to assert CO, and nurses who stated that nurses’ rights should be prioritized were more likely to report that they would assert CO in cases of abortion where the reference group was those answering “I do not know.” No other factor was significantly associated with the nurses’ intention to assert CO.

**Table 4:** Factors relating to the intention to assert conscientious objection (N = 167)

	Odds ratio	95% Confidence interval	*p* value
Age (years)	1.025	.989–1.062	.184
Gender			
Female	.626	.216–1.817	.389
Male	Reference		
Marital status			
Married	1.286	.690–2.399	.429
Single	Reference		
Clinical experience (year)	1.025	.989–1.061	.173
Clinical role			
Recovery	1.280	.533–3.070	.581
Anaesthesia	.990	.403–2.434	.983
PA/SA	.990	.295–3.325	.987
Op room	Reference		
Religion			
Protestant	2.209	1.073–4.549	.032
Catholic	1.286	.522–3.164	.584
Buddhist	.000	.000	.999
No religion	Reference		
Religion (importance)	1.380	1.038–1.835	.027
Awareness about CO			
Fully aware/aware	1.917	.973–3.779	.060
Fully unaware/unaware/neither aware nor unaware	Reference		
Resolving conflicts between nurses’ right to CO and patients’ right to receive abortion care			
Nurses’ rights	3.797	1.102–13.078	.034
Patient’s rights	.526	.188–1.475	.222
Do not know	Reference		

*CO* conscientious objection

## Discussion

This study explored perioperative nurses’ attitudes towards the legalization of CO to induced abortion, as well as their intentions to assert objector status. Prior to the legalization of abortion in the formal health care system, this study investigated nurses’ views on CO.

In this study, 45.8% supported legislation granting healthcare professionals CO status, while 22.9% opposed it. In fact, prior to this survey, most nurses (71.3%) were unaware of healthcare professionals’ CO. Logistic regression analysis showed that awareness about CO prior to this survey was significantly associated with support for the legislation. Most of the nurses in this study were unfamiliar with the issue of CO. More generally, South Koreans in the nursing profession have not actively discussed this issue. Within the nursing profession, discussions about issues regarding nurses’ CO and other related matters such as practice, policy, education, and management, are needed.

This study also asked about nurses’ view on the conflict between nurses’ right to CO and patients’ right to health care service for an abortion. The majority (68.7%) answered that patients’ rights to health care take priority, whereas 21.1% answered that nurses’ CO takes priority. In a previous study, 75.7% of nurses answered that patients’ rights to health care choices took precedence, while 24.3% answered that nurses’ CO should take precedence [23]. This is a similar trend to that in our study, which demonstrated that nurses were more than three times as likely to report that patients’ rights take priority. In our study, this was significantly associated with support for this legislation, as well as the nurses’ willingness to refuse to participate in providing abortion care. Respondents who reported that the nurses’ rights should take priority were more likely to support this legislation and to be willing to refuse to participate in providing abortion care than were those who answered “I do not know.”

There have been debates about whose rights should take precedence. Some insist that health care professionals’ right to CO should take precedence to protect their basic human rights and freedom of conscience and to prevent compromising their moral integrity and ethical well-being [16]. In addition, whether abortion is even medically beneficial to women’s health in the majority of cases is fiercely contended [24]. In contrast, others insist that health care providers’ right of conscience should not take precedence over patients’ conscience, health, and life [12]. Because it is not prima facie obvious, balance between these two competing perspectives is important in policy making. Some countries allowing CO have policies in place to reduce difficulties that women face in seeking abortion care services [9]. In other countries that prohibit CO, almost all health care providers who object to abortion choose to work in areas that are not involved in this procedure so that they can avoid moral distress while fulfilling the requirements of their professional role [12]. In South Korea, the effects of CO to abortion on health care professionals, the health care system, and women’s health needs active social discussion.

Regarding the refusal to provide abortion care, 42.5% of the nurses showed a willingness to refuse to participate in an abortion case if CO was permitted. In Davis et al., 66.4% of nurses reported they were likely to assert CO in cases that went against their religious or moral beliefs [23]. Other research has shown comparatively lower percentages. In a study by Nieminen et al., 3.5–14.1% of Finnish nursing and medical students, nurses, and physicians intended to assert CO to induced abortion [25]. Moreover, Nordstrand et al. reported that 14.7–18.5% of Norwegian medical students would be willing to refuse abortions in situations based on the length of the pregnancy and the motivation for seeking an abortion [26]. One reason for the varying rates in these studies might be that they were performed in the context of differing social reasons and health care systems. Another reason could be a measurement issue, as how the questions were asked differed between studies. Thus, it is difficult to compare the numbers directly.

Nurses’ assertion of CO to protect their moral integrity could cause problems such as discrimination against patients, limitations to patients’ access to care, difficulties in nursing staff management, and an increased workload for colleagues [15]. The number of nurses per 1,000 people in South Korea was 6.9 in 2017, which was lower than the average of 8.8 for all OECD countries and much lower than those of the top three countries (Norway, Switzerland, and Iceland with 17.7, 17.2, and 14.5, respectively) [27]. Nursing shortages and high turnover rates are issues in South Korea [28]. The Nursing shortage itself negatively affects patients’ access to care. Within the context of a national nursing shortage, nurses’ CO may have more of an effect on Korean women’s ability to receive appropriate care and may place a greater burden on managers and colleagues than it does on women in other countries that have more nurses per capita. Given nursing shortage, nurses’ ethical decision may impact patients’ access to beneficent care and nurses’ level of stress differently [29].

Although a national nursing shortage may affect the negative outcomes of nurses’ CO, certain strategies may reduce these negative outcomes. If a health care institution has a sufficient number of nurses who are non-objectors, those who object may more easily refuse when experiencing moral distress without burdening their colleagues or limiting patients’ access to care. Nurses’ CO is closely related to the issue of staffing in each institution. To protect nurses’ ethical beliefs without affecting patients’ access to care, hospital administrators and nurse managers need to make efforts to maintain a sufficient number staff and have an effective nursing staffing system in place. Moreover, ethical climates in the workplace play an important role in preventing negative outcomes from nurses’ behaviour based on their conscience [30].

On the other hand, prohibiting CO may also negatively affect patients’ health. In this study, four out of ten nurses reported their intention to CO. If CO is prohibited, nurse objectors may leave their job to avoid abortion, which would worsen the nursing shortage in abortion care. Furthermore, if they do not leave their job, they may experience negative feelings such as burnout, fatigue, anxiety, and frustration with moral distress, which could affect patients’ health and safety [15]. Therefore, considering nurses’ CO requires a more judicious approach for providing beneficial care to patients.

Of the respondents, 47.3% stated that they had a religion. Having a religion was a significant predictor of nurses’ intention to assert CO in this study. This is consistent with the findings of previous studies. In the Strickland study of medical students in the UK, Muslim and non-Muslim students had differing rates of intention to assert CO [31]. In Nordstrand et al., both perceiving religion to be important and having a religion were significantly related to medical students’ willingness to assert CO to abortion [26]. In this study, nurses who perceived religion as important were more likely to refuse to provide abortion care. Moreover, in Italy, where the Catholic Church has a greater influence on society, 68.4% of gynaecologists are conscientious objectors [32].

Regarding the type of religion, protestantism was statistically associated with nurses’ intention to assert CO, and the odds ratio for nurses who were Protestants was 2.209 (1.073–4.549; *p* = 0.032) compared with those who had no religious affiliation. On the other hand, Catholicism and Buddhism were not statistically significant factors. Among the nurses, 28.1% were Protestants, 15.0% were Roman Catholic, and 4.2% were Buddhist. In the Census Data, 43.9% of the South Korean population has a religion, and Korea’s three major religions—Protestantism, Buddhism, and Roman Catholicism—account for 19.7%, 15.5%, and 7.9% of the population, respectively [33]. In this study, the percentage of Buddhists is lower than in the general population because Buddhism is most popular among the elderly population.

Prior to the Constitutional Court ruling, the Protestant and Catholic churches of South Korea strongly expressed their official positions in favour of continuing the law banning abortion, while the Buddhists remained neutral. Specifically, the legislation of the Catholic Church is strongly against abortion [17], and the Catholic Church of South Korea demonstrated its strong opposition to abortion with a cardinal announcement and a signature-gathering campaign that reached more than one million people. Also reflecting the Catholic Church’s position, previous studies have revealed Catholic health care providers’ refusal to provide abortion care [17, 32]. However, in this study, there was no significant difference between the willingness to assert CO of Catholic nurses and nurses without a religion. The small sample size collected in the hospital could be one reason that this study does not show the influence of Catholicism. Another reason could be that Catholic nurses may have a somewhat different attitude than that of the general Catholic population because of other factors, such as professional duty. This study did not include specific questions to gather information on how the legislative framework of Catholicism impacted nurses’ intention to CO. Further research is needed to explore how nurses’ belief as Catholics increases their potential to become conscientious objectors.

This study has some limitations. First, this study used the reference of pregnancy at 22 weeks’ duration when asking about ethical dilemmas and CO; therefore, thoughts about various pregnancy durations could not be examined. Further research is needed to explore nurses’ perceptions about other pregnancy durations such as 12 and 16 weeks. Second, this study included perioperative nurses in a large hospital. However, medical abortions are more frequently provided internationally, and nurses involved in that procedure may experience a different type of stress to those involved in surgical abortions [34]. In South Korea, as abortion is legalized, the number of medical abortions will increase. Therefore, further research needs to include the perceptions of nurses in obstetrics and genecology clinics where most medical abortions are performed. Finally, this study was performed at one hospital. This limits the ability to generalize the results thereof.

## Conclusion

This study provides information for further discussions of nurses’ CO. Four out of ten nurses reported their intention to refuse to provide abortion care. The number of nurses who supported a CO clause in the abortion law was double those who opposed it. Furthermore, the majority answered that patients’ rights to health care take priority over nurses’ right to refuse it. Nursing leaders, researchers, and educators should appeal to nurses and involve them in formulating national health care policies that balance nurses' right to maintain their moral integrity with women’s rights to nondiscrimination and appropriate care. The nursing profession should seriously consider whether insisting on nurses’ right to CO is needed and should be actively involved in the process of determining the new abortion law and related policies.

As shown in this study, a considerable number of nurses want to refuse their involvement in an abortion. If CO is prohibited as per the current position of the Ministry of Health and Welfare, strategies need to be prepared to reduce these nurses’ moral distress in providing abortion care and for preventing a nursing shortage in this field. On the other hand, even if CO is legally protected, some nurses at health care institutions with insufficient nurse manpower may have difficulty in asserting their CO. Countermeasures for this issue should be prepared. Nurses’ ethical well-being is necessary in terms of providing high-quality nursing care and in ensuring patients’ safety.

This study furthers our understanding of nurses’ CO, as it is the first empirical study on this issue in South Korea. This study is especially timely and needed as we are entering a new era of legalized abortion in South Korea. More research should be performed to provide knowledge for nursing practice. In addition, because abortion has been banned for a long time, nursing education has rarely dealt with ethical issues including CO, which is why many nurses in this study were unaware of CO to abortion. Nursing education including the undergraduate curriculum and continuing education should include material dealing with the ethical issues of abortion and related CO.

## Data Availability

The dataset supporting the conclusions is available from the corresponding author on reasonable request.

## Abbreviations

CO: Conscientious objection
SD: Standard deviation
OR: Odds ratio
CI: Confidence interval
